# Comparison of chromosomal microarray and karyotyping in prenatal diagnosis using 491 amniotic fluid samples

**DOI:** 10.1097/MD.0000000000040822

**Published:** 2024-12-06

**Authors:** Ying Yang, Xiaowen Jiang

**Affiliations:** a Shangrao City Maternal and Child Health Hospital, Shangrao, China.

**Keywords:** chromosomal microarray, chromosome abnormality, copy number variation, karyotyping, prenatal diagnosis

## Abstract

This study was aimed to investigate the performance of chromosomal microarray analysis (CMA) in prenatal diagnosis compared with traditional karyotyping analysis. Both CMA and karyotyping analyses were performed to detect the karyotypes in the amniotic fluid of 491 pregnant women who got prenatal diagnosis at the Center of Prenatal Diagnosis of Shangrao (China) during January 2019 to April 2021. After excluding 2 samples in the CMA analysis and 2 samples in the karyotyping analysis which were failed in detection, the remaining 487 amniotic fluid samples were detected. Both CMA and karyotyping analyses identified 22 cases of aneuploidy chromosome abnormalities, including trisomy 21 (10 cases), trisomy 18 (4 cases), sex chromosome abnormality (5 cases), and other chromosome abnormalities (3 cases). In addition, CMA and karyotyping analyses found 8 cases of fetal chromosomal imbalance. Interestingly, abnormal results were detected by CMA analysis in 10 cases whose results were normal by karyotype analysis. Furthermore, 23 cases of copy number variation (CNVs) with variation of unknown clinical significance (VOUS) were detected by CMA, which accounted for 4.68% (23/491) in all cases. However, CMA was not able to accurately identify some complex karyotypes and mixed chimeras, including 2 cases of chimeras, 4 cases of balanced translocations, 4 cases of pericentric inversions, and 8 cases of other chromosome polymorphisms, indicating karyotyping analysis was superior to detect these chromosome abnormalities compared with CMA analysis. CMA was better in detecting the fracture sites, microduplication and microdeletion with definite pathogenicity, and CNVs with VOUS compared with karyotype analysis.

## 1. Introduction

Prenatal diagnosis is an examination item which can identify the chromosome abnormalities of embryo or fetus before birth, including aneuploidy, microduplication, microdeletion, copy number variation (CNVs), and other chromosome abnormalities.^[1]^ With the development of prenatal diagnostic techniques, there are several techniques have been used to perform prenatal diagnosis, such as karyotyping, fluorescence in situ hybridization (FISH), chromosomal microarray analysis (CMA), Next Generation Sequencing (NGS), and other techniques.^[1]^ The short detection time is the main advantage of FISH. However, FISH is unable to comprehensively reflect all the information of chromosomes.^[2]^ Besides, NGS can screen all of the DNA information to discover chromosome abnormality, but it is too expensive to be widely used.^[3]^ Currently, karyotyping is still 1 of the most widely used techniques. And an emerging application of CMA in prenatal diagnosis occurs. However, the advantages and disadvantages of these 2 techniques need further study.

The traditional chromosome G-banding karyotyping technique is the “gold standard” for prenatal diagnosis, which can analyze the number and structural abnormalities of fetal chromosomes.^[4]^ However, being affected by the long culture cycle, success rate of cell culture, low resolution, and specimen quality, karyotyping analysis is not conducive to rapid diagnosis.^[5]^ In addition, karyotyping is unable to find out the microdeletion and microduplication of fragments (<3–5 Mb) on chromosome.^[6]^ Furthermore, karyotyping can’t accurately locate the origin of additional marker chromosome.^[7]^ So, it is difficult to detect the genetic causes of fetal chromosome abnormalities only by G-banding karyotype analysis.

Chromosomal microarray analysis, also known as molecular karyotype analysis, can screen the whole genomes. In particular, it has advantages in detecting unbalanced rearrangements such as microdeletions and microduplications (<100 Kb) of chromosomes.^[8]^ Compared with G-banding karyotype analysis, CMA has better resolution, higher automation, and faster detection cycles.^[9]^ In addition, CMA can accurately locate the source of abnormal fragments that cannot be identified by karyotype analysis at the molecular level.^[8]^ Furthermore, meta-analysis reveals that CMA is more sensitive than karyotype analysis, increasing the detection rate of chromosome abnormalities and elevating the detection rate of CNVs with variation of unknown clinical significance (VOUS).^[10]^ However, CMA can’t detect chromosome translocation, inversion, polymorphism, heterochromatin, etc,^[11]^ indicating each method has its advantages and disadvantages.

In order to explore the advantages and disadvantages of CMA and traditional chromosome G-banding karyotyping analyses in prenatal diagnosis, we performed these 2 methods in amniotic fluid samples collected from 491 pregnant women and compared the results of CMA and karyotyping. This study aimed to analyze the respective advantages of CMA and karyotyping analyses. And we hope to provide a more appropriate way for prenatal diagnosis.

## 2. Methods

### 2.1. Subjects

This research was approved by Medical Ethics Committee of Shangrao Maternal and Child Health Hospital with the number of SRFB20190110001. A total of 491 pregnant women who got prenatal diagnosis at the Center of Prenatal Diagnosis of Shangrao (China) during January 2019 to April 2021 were recruited for this retrospective study. All of the participants have signed the informed consents. Then the amniotic fluid samples were collected to conduct karyotyping and chromosomal microarray assays. And the inclusion and exclusion criteria were showed as in Table 1. And the detailed information of patients was available in Table 2.

**Table 1:** Inclusion and exclusion criteria of subjects.

Inclusion criteria
Age (18–44)
The gestational age ranged from 16 to 29 weeks with sufficient amniotic fluid and cells
Abnormal ultrasound
Advanced age
High risk in Down syndrome
The results of noninvasive prenatal diagnosis were high or closed to critical risk
Adverse pregnancy history or family history of genetic disease for 1 of the spouses
Exclusion criteria
Failed in amniotic-fluid-cell culturing
Failed in CMA detection
Abortion or threatened abortion
Infection
Disturbance of blood coagulation
Damage or dis-function in visceral organs
Malnutrition, hypoglycemia, or anemia

**Table 2:** Basic information of subjects.

Variables	Inclusion (n)	Exclusion (n)	CMA abnormality (n)	Karyotyp abnormality (n)
Age (18–44)	491	0	63	45
Gestational weeks (16–29)	491	0	63	45
Abnormal ultrasound	201	0	16	7
High risk in Down syndrome	169	0	12	3
Noninvasive prenatal diagnosis for high or borderline risk	56	0	11	18
Adverse pregnancy history	27	0	4	2
Family history of genetic disease for 1 of the spouses	15	0	3	4
Advanced age	70	0	8	0
Abnormal amniotic fluid	6	0	2	0
Use drugs or alcohol during pregnancy	6	0	0	2
in vitro fertilization	2	0	0	0
Two or more abnormities	38	0	7	9
CMA detection failure		2		
Failed in amniotic-fluid-cell culturing		2		

Inclusion criteria: aged 18 to 44 years, the gestational age ranged from 16 to 29 weeks with sufficient amniotic fluid and cells, abnormal ultrasound, advanced age, high risk in Down syndrome, the results of noninvasive prenatal diagnosis were high or closed to critical risk, adverse pregnancy history or family history of genetic disease for 1 of the spouses.

Exclusion criteria: failed in amniotic-fluid-cell culturing, failed in CMA detection, abortion or threatened abortion, infection, disturbance of blood coagulation, damage or dis-function in visceral organs, malnutrition, hypoglycemia, or anemia.

### 2.2. Karyotyping

Traditional chromosome karyotyping analysis was performed by 2 individuals with 26 or 6 years of working experience. Amniotic fluid samples (20 mL per sample) were collected via amniocentesis intraperitoneally. After centrifugation at 2000 rpm for 5 minutes, 0.5 mL amniotic fluid was retained, then 5 mL culture medium was added to resuspend the cells. The cells were cultured in a cell culture flask at 37 °C in a cell incubator with 5% CO_2_ for 5 to 7 days. Then the culture medium was renewed. And the cells were harvested after 8 to 10 days. After hypotension, pre-fixation, fixation, and repeated fixation, the cells were attached to the slides and stained with Giemsa stain for 2 minutes. Then the karyotype were visualized by using the optical microscope with automatic scanning analyzer.

### 2.3. Chromosomal microarray analysis

Collect amniotic fluid samples from patients and preprocess the samples, including DNA extraction and purification, to obtain high-quality DNA templates. Fragment the extracted DNA samples and add adapter sequences to construct sequencing libraries. Utilize high-throughput sequencing technology to sequence the libraries, yielding a large amount of DNA sequence information. Compare the sequencing results with a reference genome to identify variations such as microduplications and microdeletions on the chromosomes. And CMA was carried out by 3 researchers in the genetic laboratory of maternal and child health care hospital of Jiangxi province. The CMA analysis could identify the chromosome microduplication, microdeletion, and loss of heterozygosity which were <100 kb in the whole genome.^[12]^

### 2.4. Copy number variation

CNV classification criteria: according to the quantitative scoring classification of the ACMG + ClinGen guidelines: pathogenic variant (P), likely pathogenic variant (LP), variant of uncertain significance, likely benign variant (LB), and benign variant (B).

## 3. Results

**Figure 1.**
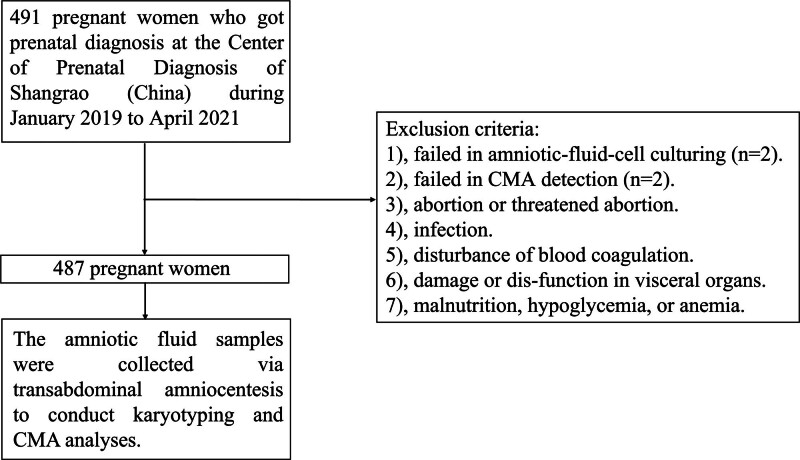
Flow chart of subject screening.

A total of 491 amniotic fluid samples were involved in this study. After excluding 2 samples in the CMA analysis and 2 samples in the karyotyping analysis which were failed in detection, the remaining 487 amniotic fluid samples were detected by both CMA and karyotyping analyses (Fig. 1). As shown in Table 3, we found the coincidence rate of the results of CMA and karyotyping analyses was 90.14% (439/487). And the detection rate of chromosomal abnormality by CMA analysis (12.94%, 63/487) was higher than that by karyotyping analysis (9.24%, 45/487). It would be 16.02% (78/487) if the chromosomes were abnormal by CMA and/or karyotyping analyses. The results of CMA and karyotyping analyses indicated 23 cases of aneuploid chromosome abnormality, included trisomy 21 (10 cases), trisomy 18 (4 cases), 47, XXY (2 cases), 47, XYY (2 cases), 47, XXX (1 case), 45, X (1 case), 46, XN, +21, rob (14; 18) (1 case), 47, XN, +21 [26]/46, XN [74] (1case), and 46, XY [6]/46, XY, +21 [44] (1 case).

**Table 3:** Fetal chromosome aneuploidy detected by Karyotype and CMA analyses.

Category No.	Karyotype results	No. of chromosomal abnormality	CMA detection results	Disease name
1	47,XN,+21	10	arr[hg19](21) × 3	Trisomy 21
2	47,XN,+18	4	arr[hg19](18) × 3	Trisomy 18
3	47,XXY	2	arr(X) × 2,(Y) × 1	Klinefelter syndrome
4	47,XYY	2	arr(X) × 1,(Y) × 2	Super-man syndrome
6	45,X	1	arr[hg19](X) × 1	Turner syndrome
7	46,XN,+21,rob(14;18)	1	arr[hg19](21) × 3	Trisomy 21, Robertsonian translocation
8	47,XN,+21 [26]/ 46,XN[74]	1	arr(21) × 2 to 3	Trisomy 21, chimera
9	46,XN[6]/46,XN,+21[44]	1	arr[hg19](21) × 3	Trisomy 21, chimera
10	47,XXX	1	Arr(X) × 3	Superfemale syndrome

As revealed by the category No. 7 in Table 3, the CMA analysis could not identify whether the chromosomal abnormality of trisomy 21 was Robertson translocation or standard types, indicating the CMA analysis was not suitable to detect chromosomal structural abnormality and relative hereditary information of chromosome imbalance. Interestingly, abnormal results were detected by CMA analysis in 10 cases whose results were normal by karyotype analysis. The fragment sizes were from 577 Kb to 3.79 Mb in the identified microduplication and microdeletion. According to the CNV classification standard in American College of Medical Genetics and Genomics (ACMG) guidelines, all of these chromosomal abnormalities detected by CMA analysis were pathogenic variations. The rate of definite pathogenicity detected by CMA was 2.05% (10/487) higher than that detected by karyotype analysis (Table 4). These results revealed CMA analysis could identify microduplication and microdeletion with definite pathogenicity.

**Table 4:** The abnormal results detected by CMA of 10 cases with normal karyotype detected by karyotype analysis.

Chromosomal variation segment	Variation type	Fragment length	Variation classification	Indications for prenatal diagnosis
16p11.2	dup	557 Kb	Pathogenic	High Risk in Down syndrome screening
5q11.2	del	788 Kb	Pathogenic	Oligohydramnios
22q11.21	dup	2.54 Mb	Pathogenic	Ultrasound abnormality
Xp22.31 (4 cases)	del	1.68 Mb	Pathogenic (male)	High risk in Down syndrome screening (2); ultrasound abnormality (1); NIPT anomaly (1)
17p11.2	del	3.79 Mb	Pathogenic	NIPT anomaly
1q21.1q21.2 (2 cases)	dup	1.30 Mb	Pathogenic	NT:3.01 mm (1); advanced maternal age (1)

Furthermore, as shown in Table 5, both traditional chromosome karyotyping analysis and CMA analysis found 8 cases of fetal chromosomal imbalance. The results revealed compared with traditional karyotype analysis, CMA detection could more accurately identify the fracture sites, together with the microduplication and microdeletion which could not be detected by traditional karyotype. Besides, according to the copy number interpretation guidelines of ACMG and the CNV reporting principles in our laboratory, 23 cases of CNVs with VOUS were detected by CMA analysis, which accounted for 5.62% (23/409) in the cases of which the chromosomes were normal by CMA and/or karyotyping analyses. However, the CNVs in these 23 cases were undetected by karyotype analysis (Table 6). These results indicated CMA analysis was better in identifying the fracture sites, microduplication, microdeletion, and CNVs compared with karyotyping analysis.

**Table 5:** Karyotype analysis and CMA results of 8 cases of chromosome imbalance structural abnormality.

Karyotype analysis	Variation segment	Fragment length	Variation type	Variation classification	Indications for prenatal diagnosis
46,XN,psu dic(10;18) (p15.1;p11.21)	10p15.315.1	3.95 Mb	del	Pathogenic	NIPT anomaly
	18p11.32p11.21	15.03 Mb	del	Pathogenic	NIPT anomaly
46,XN,del(11)(p12p11.2)	11p12p11.2	9.18 Mb	Micro-del	Pathogenic	NIPT anomaly
46,XN,del(2) (q22.3,23.2)	2q22.323.2	6.23 Mb	del	Pathogenic	Advanced maternal age
46,XN,del(9),del(9)(p24.2,24.3)dup(9) (p24.2q13)	9p24.2,24.3	2.32 Mb	del	Like pathogenic	Ultrasound abnormality
	9p24.2q13	65.67 Mb	Micro-del	Pathogenic	Ultrasound abnormality
46,XN,del(X)(p22.33p22.13)	Xp22.33p22.13	18.49 Mb	Micro-del	Pathogenic	Advanced maternal age; NIPT anomaly
46,XN,del(9)(p24.3p23)	9p24.3p23	9.26 Mb	del	Pathogenic	Ultrasound abnormality
46,XN,del(6)(q25.3q27)	6q25.3q27	13.83 Mb	del	Pathogenic	Ultrasound abnormality
46,X,del(X)del(X) (p11.2)add(X)(P11.2)	Xp22.33p11.22	52.62 Mb	del	Pathogenic	NIPT anomaly

**Table 6:** Copy number variation of unknown clinical significance in CMA detecting.

Variation segment	Variation type	Fragment length	Variation classification	Indications for prenatal diagnosis
4p16.3	dup	375Kb	VOUS	Family history of Down syndrome
1p21.2	dup	2.98Mb	VOUS	High risk in Down syndrome screening
16p13.11	dup	827Kb	VOUS	High risk in Down syndrome screening
6q12	dup	3.39Mb	VOUS	High risk in Down syndrome screening
16p13.11p12.3	dup	2.96Mb	VOUS	High risk in Down syndrome screening
5q34q35.1	dup	1.35Mb	VOUS	High risk in Down syndrome screening
2q21.1	del	523Kb	VOUS	Advanced maternal age
17q11.2q21.33	Homozygous for large fragments	22.64Mb	VOUS	Advanced maternal age
16q24.1q24.2	dup	2.64Mb	VOUS	High risk in Down syndrome screening
4q25	Micro-dup	4.3Mb	VOUS	Ultrasound abnormality
9q22.2p21.3	Micro-del	1.72Mb	VOUS	Ultrasound abnormality
3p26.3	del	1.93Mb	VOUS	NIPT anomaly
6q27	Micro-dup	1.38Mb	VOUS	Ultrasound abnormality
7q31.33	Micro-dup	1.96Mb	VOUS	Ultrasound abnormality
15q11.2	del	855Kb	VOUS	NT:3.2 mm
6q12q14.1	ROH	10.45Mb	VOUS	Ultrasound Abnormality
10q11.22q11.23	Micro-dup	5.52Mb	VOUS	Self karyotype is 45,X/46,XX mosaic
Xq12q21.2	dup	9.3Mb	VOUS	NIPT anomaly
6p22.3p21.3	ROH	10.2Mb	VOUS (increased autosomal recessive risk)	Advanced maternal age
2q12.3q13	Micro-del	1.11Mb	VOUS	Ultrasound abnormality
18q11.2q12.3	ROH	19.23Mb	VOUS (increased autosomal recessive risk)	High risk in Down syndrome screening
3p26.3	del	1.93Mb	VOUS	NIPT anomaly
16p12.2	del	542Kb	VOUS	Advanced maternal age; high risk in Down syndrome screening

In order to comprehensively unveil the advantages and disadvantages of CMA analysis, we further found out the chromosomes with normal CMA results that were identified abnormally by karyotyping analysis. As shown in Table 7, these chromosomes included 2 cases of chimeras, 4 cases of balanced translocations, 4 cases of pericentric inversions, and 8 cases of other chromosome polymorphisms, indicating karyotyping analysis was superior to detect these chromosome abnormalities compared with CMA analysis. According to the case of NO.10 in Table 7, we found CMA analysis could not identify low level of chimerism. However, the case of NO.11 with high level of chimerism also could not be identified by CMA analysis. Further detecting the case of NO.11 by FISH, we revealed the chromosome of No. 11 was mixed chimeras with 3 karyotypes, including 45, X [30] (56%), 46, XY [30] (12%), and another karyotype, 47, XYY (22%), which was not identified by karyotyping analysis. These results indicated it was better to apply different methods to detect samples with complex karyotypes and mixed chimeras.

**Table 7:** The abnormal results detected by karyotype analysis of 18 cases with normal karyotype detected by CMA analysis.

Category No.	Karyotype	Case No.	CMA	Chromosome description
1	46,XN,inv(6)(p11.2p25)	1	arr(1–22) × 2	Pericentric inversion of chromosome 6
2	46,XN,inv(9)(q11q13)	2	arr(1–22) × 2	Pericentric inversion of chromosome 9
3	46,XN,inv(Y)(p11.2q11.2)	1	arr(1–22) × 2	Pericentric inversion of chromosome Y
4	46,XN,16qh+	2	arr(1–22) × 2	Increased length of heterochromatin region of chromosome 16
5	46,XN,9qh+	2	arr(1–22) × 2	Increased length of heterochromatin region of chromosome 9
6	46,XN,22ps+	1	arr(1–22) × 2	Increased length of satellite of chromosome 22
7	46,XN,15ps+	1	arr(1–22) × 2	Increased length of satellite of chromosome 15
8	46,XN,21pstk+	1	arr(1–22) × 2	Increased length of satellite stalk of chromosome 21
9	46,XN,22pstk+	1	arr(1–22) × 2	Increased length of satellite stalk of chromosome 22
10	47,XXY[5]/46,XY[41]	1	arr(1–22) × 2	47, XXY and 46, XY formed chimera
11	45,X[30]/46,XY[30]	1	arr(1–22) × 2	45,X and 46,XY formed chimera
12	46,XN,t(9;10)(p22;q23)	1	arr(1–22) × 2	Balanced translocations on chromosomes 9 and 10
13	46,XN,t(4;18)(p15.3;q13.2)	1	arr(1–22) × 2	Balanced translocations on chromosomes 4 and 18
14	46,XN,t(5;10)(q13;q12)	1	arr(1–22) × 2	Balanced translocations on chromosomes 5 and 10
15	46,XN,t(1;13)(q32;q14)	1	arr(1–22) × 2	Balanced translocations on chromosomes 1 and 13

## 4. Discussion

CMA and karyotyping each have their own advantages in genetic testing: CMA boasts higher resolution, capable of detecting more subtle chromosomal abnormalities such as deletions and duplications of chromosomal segments. Karyotyping primarily detects large-scale abnormalities in chromosome number and structure, such as those seen in Down syndrome. CMA is suitable for detecting CNVs of chromosomal imbalances at the whole-genome level, particularly advantageous for detecting genomic imbalances such as microdeletions and microduplications. Karyotyping can visually display the overall morphology of chromosomes but has lower resolution, unable to detect “microdeletions” or “microduplications.” CMA has a relatively shorter detection cycle, providing results more quickly. Karyotyping requires cell culture, and if cell culture fails, the test fails. CMA is relatively automated in its operation, reducing the subjectivity of manual analysis. Karyotyping is purely manually analyzed, and result interpretation is influenced by the analyst’s experience and subjective judgment. CMA is often used in prenatal genetic diagnosis, such as testing aborted fetuses or fetal chromosomes after invasive prenatal testing. For patients with intellectual disability and/or developmental delay of known causes, multiple malformations of known syndromes, and autism spectrum disorders, CMA can serve as a first-line detection method. When determining whether fetal chromosomal issues are de novo or sporadic, polymorphic or pathogenic, CMA offers higher accuracy. Karyotyping is applicable for diagnosing genetic diseases caused by chromosomal abnormalities, studying kinship, exploring species evolution, and other scenarios. It can determine whether a fetus has chromosomal abnormalities, especially suitable for pregnant women after 18 weeks of gestation. CMA can detect changes in the quantity of genetic material in samples but cannot detect balanced chromosomal structural abnormalities such as translocations and inversions. Additionally, they cannot detect low-level mosaicism. Karyotyping is typically conducted on prospective parents, while CMA is used for invasive prenatal testing on aborted fetuses or fetal chromosomes (samples such as chorionic villus, amniotic fluid, umbilical cord blood, or fetal tissue).

In this study, we explored the advantages and disadvantages of CMA and karyotype analyses in prenatal diagnosis. Our results revealed CMA was better in detecting the fracture sites, microduplication and microdeletion with definite pathogenicity, and CNVs with VOUS compared with karyotype analysis. However, CMA could not identify mixed chimeras, balanced translocations, pericentric inversions, and chromosome polymorphisms which were complicated. Our study showed application of these 2 methods would be useful to better understanding the chromosome abnormalities.

CMA has been commonly used in prenatal diagnosis. Previous study revealed the abnormality rate detected by karyotype analysis was 5.06%. And the detection rate of chromosomal abnormality of CMA was 9.14%, which was 4.08% higher than karyotype analysis.^[13]^ Another study also showed the abnormal rate of chromosome detected by CMA was increased by 3.8% compared with karyotype analysis.^[14]^ Similar to these studies, we found the detection rate of chromosomal abnormality by CMA analysis was 3.7% higher than that by karyotyping analysis. Actually, CMA was the preferred method for prenatal diagnosis when there were multiple abnormalities in ultrasound.^[15]^ Karyotyping was flawed in detecting the microdeletion and microduplication of fragments (< 3–5 Mb) on chromosome6. However, CMA had advantages in detecting unbalanced rearrangements such as microdeletions and microduplications (< 100 Kb) of chromosomes by screening the whole genomes8. Our study indeed revealed that CMA analysis was better in identifying fracture sites, microduplication, microdeletion, and CNVs compared with karyotyping analysis. Nevertheless, CMA was unable to replace karyotyping analysis. It has been reported CMA has no advantage in detecting chromosomal translocations and low level of chimera.^[13,16,17]^ Consistent with this study, our study also revealed CMA could not identify mixed chimeras and balanced translocations. Furthermore, the results indicated karyotyping analysis was superior to identify pericentric inversions and chromosome polymorphisms which were complicated compared with CMA.

As a high-resolution method, CMA could identify the CNVs that could not be detected by karyotyping analysis.^[8,18]^ CNVs was the cytogenetic basis of many genetic diseases. According to the characteristics of CNVs, genetic diseases could be diagnosed.^[19]^ According to the classification standard of the ACMG, the results of CNVs could be divided into 3 categories: benign CNV, CNV of VOUS, and pathogenic CNV. Distinction between pathogenic and nonpathogenic was the most critical issue.^[20]^ We found pathogenic CNVs were detected by CMA analysis in 10 cases whose results were normal by karyotype analysis. Among them, the deletion variation of Xp22.31 was related to X-linked ichthyosis.^[21]^ And 1q21.1q21.2 microduplication was associated with developmental delay and congenital heart defect.^[22]^ Actually, previous works also demonstrated CMA was better in detecting CNVs compared with karyotype analysis.^[14]^ In line with this study, we found that CMA could identify 23 cases of CNVs with VOUS, which were detected normal in karyotype analysis.

Even though the detection rate of chromosome abnormality by CMA was higher than that by karyotype analysis, G-banding karyotype analysis could detect complex karyotypes and chromosome polymorphisms such as balanced translocations, Robertson translocations, inversions and insertions that could not be detected by CMA. Currently, the effective strategy adopted by many prenatal diagnosis centers of China was the application of karyotype analysis combined with CMA for prenatal diagnosis.^[23–27]^ These 2 detection techniques were complementary in the detection of chromosomal abnormalities. Consistent with previous studies, our results also revealed combined use of CMA and karyotype analyses would increase 3.08% and 6.78% in chromosomal abnormalities detection rate, compared with that detected by CMA or karyotype analysis only.

The traditional chromosome G-banding karyotype analysis relies on the experience of the operator. In addition, this method will spend long time culturing cells. And it is not conducive to rapid diagnosis. Furthermore, karyotype analysis can’t detect chromosome microdeletion and microduplication. Compared with traditional karyotype analysis, CMA technology has better resolution and higher detection rate. What is more, CMA can accurately identify microdeletion and microduplication of chromosome. However, CMA is unable to detect mixed chimeras and balanced translocations. So, CMA and karyotype analyses are complementary in the detection of chromosome abnormalities. Our study provided clinical basis and data support for clinicians to choose suitable prenatal diagnosis methods for different purposes.

In summary, the detection rate of chromosomal abnormality of CMA was higher than karyotype analysis. Furthermore, CMA could identify the CNVs that could not be detected by karyotyping analysis. However, CMA was unable to identify mixed chimeras, balanced translocations, pericentric inversions and chromosome polymorphisms which were complicated. And combined use of CMA and karyotype analysis could comprehensively reflect the chromosomal abnormalities.

## Author contributions

**Conceptualization:** Ying Yang.

**Data curation:** Ying Yang, Xiaowen Jiang.

**Formal analysis:** Ying Yang, Xiaowen Jiang.

**Methodology:** Xiaowen Jiang.

**Writing – review & editing:** Ying Yang, Xiaowen Jiang.

**Writing – original draft:** Xiaowen Jiang.
